# The Rule of the Artery: Management of Peripheral Vascular Disease with Osteopathic Manipulative Treatment in a Low Resource Setting in Iquitos, Peru

**DOI:** 10.51894/001c.144481

**Published:** 2025-10-01

**Authors:** Makenzie Kamm

**Affiliations:** 1 College of Osteopathic Medicine Michigan State University

## 90

### INTRODUCTION

MSUCOM provides an outreach clinic in Iquitos, Peru, for one week each year. We present a case of a patient seen at this clinic with PVD secondary to chronic, uncontrolled Type 2 Diabetes Mellitus (T2DM) with an acute obstruction of blood flow to the right foot.

Peripheral vascular disease (PVD) is a circulatory disorder characterized by the narrowing, blockage, or spasm of arteries outside the heart and brain, and is most commonly found in the lower extremities, with T2DM, smoking, hypertension and hyperlipidemia attributed to disease progression. Osteopathic manipulative treatment (OMT) is a hands-on approach that identifies and addresses asymmetry, range of motion restrictions, and tissue texture abnormalities. Although not typically used in treatment of PVD, the present case suggests the potential of OMT as an adjunct form of PVD treatment.

### CASE DESCRIPTION

The patient is a 50 year old male with a 15-year history of T2DM, who experienced worsening pain, discoloration and temperature changes of the right foot, 30 hours prior to arrival to the clinic. These were determined to be signs of critical ischemia requiring urgent surgical intervention, however, this option was not available in the low resource setting. OMM was provided in the form of indirect myofascial release and balanced membranous tension directed to the right lower extremity, pelvis and respiratory diaphragm in three treatments over the course of three days. This resulted in a progressive improvement in color, temperature and pain.

### DISCUSSION/CONCLUSIONS

OMT was used in this case given the lack of prompt and adequate surgical treatment. PVD is viewed as an intravascular issue where narrowing occurs within the arteries. This case suggests that the symptoms were due to external compression from myofascial tension on the lower extremity arteries, thus presenting a novel perspective on vascular health. OMT is not a substitute for surgical intervention but appears to assist in restoration of blood flow while the definitive treatment is established. This case highlights the adaptability of osteopathic principles in global health settings, demonstrating how hands-on treatment can be a powerful adjunct in managing acute complications of chronic diseases such as PVD in low resource areas.

